# Mechanisms of Adhesion and Subsequent Actions of a Haematopoietic Stem Cell Line, HPC-7, in the Injured Murine Intestinal Microcirculation *In Vivo*

**DOI:** 10.1371/journal.pone.0059150

**Published:** 2013-03-12

**Authors:** Dean P. J. Kavanagh, Adrian I. Yemm, Yan Zhao, Jon Frampton, Neena Kalia

**Affiliations:** 1 Centre for Cardiovascular Sciences, College of Medical and Dental Sciences, University of Birmingham, Birmingham, United Kingdom; 2 School of Immunity and Infection, Institute of Biomedical Research, College of Medical and Dental Sciences, University of Birmingham, Birmingham, United Kingdom; Northwestern University, United States of America

## Abstract

**Objectives:**

Although haematopoietic stem cells (HSCs) migrate to injured gut, therapeutic success clinically remains poor. This has been partially attributed to limited local HSC recruitment following systemic injection. Identifying site specific adhesive mechanisms underpinning HSC-endothelial interactions may provide important information on how to enhance their recruitment and thus potentially improve therapeutic efficacy. This study determined (i) the integrins and inflammatory cyto/chemokines governing HSC adhesion to injured gut and muscle (ii) whether pre-treating HSCs with these cyto/chemokines enhanced their adhesion and (iii) whether the degree of HSC adhesion influenced their ability to modulate leukocyte recruitment.

**Methods:**

Adhesion of HPC-7, a murine HSC line, to ischaemia-reperfused (IR) injured mouse gut or cremaster muscle was monitored intravitally. Critical adhesion molecules were identified by pre-treating HPC-7 with blocking antibodies to CD18 and CD49d. To identify cyto/chemokines capable of recruiting HPC-7, adhesion was monitored following tissue exposure to TNF-α, IL-1β or CXCL12. The effects of pre-treating HPC-7 with these cyto/chemokines on surface integrin expression/clustering, adhesion to ICAM-1/VCAM-1 and recruitment *in vivo* was also investigated. Endogenous leukocyte adhesion following HPC-7 injection was again determined intravitally.

**Results:**

IR injury increased HPC-7 adhesion *in vivo*, with intestinal adhesion dependent upon CD18 and muscle adhesion predominantly relying on CD49d. Only CXCL12 pre-treatment enhanced HPC-7 adhesion within injured gut, likely by increasing CD18 binding to ICAM-1 and/or CD18 surface clustering on HPC-7. Leukocyte adhesion was reduced at 4 hours post-reperfusion, but only when local HPC-7 adhesion was enhanced using CXCL12.

**Conclusion:**

This data provides evidence that site-specific molecular mechanisms govern HPC-7 adhesion to injured tissue. Importantly, we show that HPC-7 adhesion is a modulatable event in IR injury and further demonstrate that adhesion instigated by injury alone is not sufficient for mediating anti-inflammatory effects. Enhancing local HSC presence may therefore be essential to realising their clinical potential.

## Introduction

Although the incidence of inflammatory bowel disorders (IBD) is rising in western countries, current treatments remain inadequate with no long-term efficacy observed. Stem cell (SC)-based cellular therapies offer promising approaches for treating a wide variety of inflammatory disorders. Recent attention has focussed on bone marrow (BM)-derived mesenchymal SCs (MSCs) primarily due to their anti-inflammatory and immunomodulatory effects and low immunogenicity [1]. However, complications such as tumour formation, their potential to differentiate into unwanted mesenchymal lineages and pulmonary entrapment following systemic infusion of these large cells has led to recent caution being urged when considering these cells for clinical use [2].

BM-derived haematopoietic SCs (HSCs) have also been shown to confer therapeutic benefit for many inflammatory disorders including in experimental models of colitis and in Crohn's patients [3]. Emerging data has demonstrated that they too can directly influence the progression of inflammation in injured tissues in a paracrine manner [4]. Indeed, human HSCs are able to secrete both pro- and anti-inflammatory growth factors and cytokines, such as transforming growth factor-β1 (TGF-β1), stem cell factor (SCF), and tumour necrosis factor-α (TNF-α) [5]. Upon stimulation with pro-inflammatory mediators, HSCs can release reparative factors, such as epithelial growth factor (EGF), fibroblast growth factor (FGF), platelet derived growth factor (PDGF) and vascular endothelial growth factor (VEGF) [6]. However, despite clinical evidence that HSCs can improve IBD, benefits are either minor or transitory. The rarity of HSCs (<0.01% of BM), along with poor tissue homing and retention, may contribute to their limited clinical utility and success [7]. If HSC therapy is to be realised, a better understanding of the basic science underlying their recruitment to injured sites is essential.

Our knowledge of the adhesive mechanisms mediating recruitment of transplanted HSCs by the injured intestinal microcirculation is limited, with studies focused primarily on homing to healthy BM [8]. HSCs exhibit a similar repertoire of surface adhesion molecules to mature leukocytes, expressing CD29 (β_1_) and CD18 (β_2_) integrins which bind to their endothelial counter-receptors, VCAM-1 and ICAM-1 respectively [9]. Recent work from our group demonstrated a critical role for the CD49d (α_4_ subunit of α_4_β_1_ integrin)/VCAM-1 pathway in mediating HSC recruitment to murine injured liver [10]. Similar interactions also mediate recruitment to BM, implicating an important role for this integrin in HSC homing [11]. However, it is not known whether CD49d is universally responsible for retaining HSCs in all injured vascular beds. This study therefore determined the molecular adhesive mechanisms governing HSC recruitment to the ischemia-reperfusion (IR) injured murine small intestine *in vivo*. To further identify if the mechanisms governing HSC adhesion within the gut were site specific, HSC trafficking within the IR injured cremaster muscle was also monitored.

Since SC surface integrins play an important part in SC-endothelial cell interactions, modulating their expression and/or affinity for endothelial counterligands might be an important approach to improve SC homing and thus potentially enhance the effectiveness of SC therapy. A variety of chemical mediators are released from inflamed tissue, including cytokines, that can activate integrins on trafficking HSCs and subsequently initiate their adhesion to microvessels [12]. This occurs in a similar manner to that described for leukocyte activation. Indeed, HSCs are well equipped with additional surface molecules such as cytokine/chemokine receptors and signaling molecules, indicating that they are highly reactive to their microenvironment. Therefore, the roles of the inflammatory cytokines, CXCL12 (stromal cell-derived factor-1α), interleukin-1β (IL-1β) and TNF-α in promoting intestinal HSC homing were also determined. Futhermore, this study investigated whether pre-treating HSCs with these cytokines prior to their systemic administration could enhance their homing efficiency to the injured gut. Given the observed experimental benefit of HSCs in IBD and the ability of HSCs to secrete anti-inflammatory cytokines, we hypothesised that the presence of HSCs within the IR injured gut would result in a dampening of the inflammatory response to injury. To assess this, endogenous leukocyte recruitment in the injured intestinal microcirculation was also monitored *in vivo*. In this report we provide novel evidence that HSCs can indeed be recruited to the injured gut, that this event is modulatable by pre-treating them with cytokines and more importantly, only by enhancing local HSC recruitment can inflammatory cell infiltration be reduced within the injured microcirculation.

## Materials and Methods

### Animals

All Male C57BL/6 mice (8–12 weeks) were used for procedures in accordance with the Animals (Scientific Procedures) Act 1986, under licence from the United Kingdom Home Office (PPL 40/3336). All procedures were performed under ketamine/xylazine anaesthesia (intraperitoneal administration). Anaesthetized animals underwent carotid artery cannulation to facilitate infusion of HSCs (HPC-7), Acridine Orange (AcrO) or administration of additional anaesthetic.

### Cells

Intravital studies monitoring HSC trafficking have been limited due to difficulties in isolating sufficient numbers for *in vivo* experimentation [13]. As a result, many HSC trafficking studies have relied heavily on HSC lines such as FDCP-mix [14]. In this study, we have utilized an immortalised HSC line, HPC-7, generated by transfecting murine embryonic SCs with the gene *LHx2* [15]. HPC-7 display many characteristics of primary HSCs, including high expression of common murine HSC markers as well as being lineage negative [15]. HPC-7 express early haematopoietic HSC transcription markers, such as SCL, c-myb, GATA-1 and PU.1 [15], and are responsive to known haematopoietic transcription factors such as Scl [16]. In addition, we have demonstrated previously that HPC-7 express adhesion molecules known to be present on primary HSCs and have previously used HPC-7 to model hepatic HSC recruitment [10]. Furthermore, a number of key adhesion molecules identified on HPC-7, including CD18 and CD49d, are present on primary KSL cells (lineage negative, c-Kit^+^, Sca-1^+^) isolated from C57BL/6 mice [17], [18]. HPC-7 cells were obtained as a gift from Prof. Leif Carlsson (University of Umeå, Sweden). HPC-7 were maintained in Stem Pro-34 SFM supplemented with the manufacturers media supplement (Invitrogen, UK), 100ng/ml SCF (Invitrogen), L-Glutamine (PAA, Somerset, UK), penicillin and streptomycin (PAA). Cells were labelled with 5 µM CFDA-SE (Invitrogen) prior to use. For clustering studies, cells were labelled with 10 µM CellTracker Orange (CTO; Invitrogen). Some HPC-7 were pre-treated for 30 minutes with 80 µg/ml of anti-mouse CD18 (GAME-46), anti-mouse CD49d (R1-2) or rat IgG control antibody (RTK2758) (Cambridge Biosciences, UK) prior to use.

### Frozen tissue static adhesion assay

Terminal ileal, jejunal or duodenal segments were isolated and snap frozen from sham and IR injured animals (45 min ischaemia; 120 min reperfusion) and sectioned (10 µm). 1×10^5^ CFSE labelled HPC-7 were applied to each section for 20 minutes and then washed with PBS to remove unbound cells. Sections were fixed in acetone and adherent cells from 10 random fields were counted microscopically and the mean obtained.

### Endothelial static adhesion assay

Murine immortalized colonic endothelial cells (ECs; gift from Dr. J. Steven Alexander, LSU-HSC, USA) were cultured to confluence on gelatin-coated tissue culture plates (Nunc, USA). At confluence, monolayers were treated for four hours with 100 ng/ml TNF-α (Peprotech, London, UK). CFSE labeled cells were pre-treated with either PBS (Invitrogen) or 100 ng/ml of either CXCL12, TNF-α or IL-1β (Peprotech) for one hour. HPC-7 were subsequently washed and endothelial monolayers exposed to 1×10^5^ HPC-7 for 20 minutes. Wells were washed with PBS and fixed with 2% glutaraldehyde in PBS (15 minutes; 37°C). Adherent cells from 5 random fields were counted using fluorescent microscopy and the mean obtained.

### Flow Cytometry

Changes in the surface expression of CD18 (M18/2) or CD49d (R1-2) (eBioscience, CA, USA) were examined using flow cytometry. 1×10^5^ HPC-7 were pre-treated for 4 hours with PBSA (PBS with 0.1% bovine serum albumin) or 20 ng/ml of either CXCL12, TNF-α or IL-1β. Cells were then fixed with 10% formalin (Sigma-Aldrich) and then resuspended in 100 µl PBSA containing 5% FBS (PAA). Cells were incubated with primary antibody at 1∶50 prior to flow cytometry (FACSCalibur, BD, NJ, USA) and analysed with Summit (DakoCytomation, CO, USA). Expression of cytokine receptors was similarly assessed by incubating HPC-7 with PE-conjugated anti-CD120a (TNFR1; 55R-285, Biolegend, USA), PE-conjugated anti-CD120b (TNFR2; TR75-89, Biolegend) or Alexa 488-conjugated anti-CD121a (IL-1RI; JAMA-147; AbD Serotec, UK). For CXCR4, cells were incubated with rat anti-mouse CXCR4 primary (247506; R&D Systems, UK) and FITC-conjugated goat anti-rat IgG secondary (Poly4054; BioLegend).

To examine the expression of adhesion molecules on KSL cells, BM was isolated from C57BL/6 mice (5-8 mice per repeat). BM was depleted for Lin^+^ cells using a magnetic bead based Lineage depletion kit in accordance with the manufacturer's instructions (Lineage cell depletion kit, Miltenyi Biotec, UK). Lineage depleted cells were subsequently labelled with PE-conjugated Sca-1 (Ly6A/E, clone D7; eBioscience), PE/Cy5-conjugated c-Kit (CD117, clone 2B8; eBioscience) and either FITC-conjugated IgG, FITC-conjugated CD18 (M18/2) or FITC-conjugated CD49d (R1-2). In some experiments, intracellular antigens were detected by permeabilization. Lin^−^ cells were with fixed in 10% Neutral Buffered Formalin (Sigma-Aldrich) for 10 minutes at room temperature. Subsequently, cells were washed and resuspended in 0.1% Triton X-100 in PBS for 10 minutes before being washed twice again. Cells were analysed using a BD FACSCalibur device. Cells were gated for Sca-1 and c-Kit positivity and the adhesion molecule expression of these cells indentified. Expression is represented as fold increase in mean fluorescence intensity compared to IgG.

### Intravital imaging of small intestine

To induce intestinal IR injury, the superior mesenteric artery was clamped for 45 minutes with reperfusion initiated following clamp removal. Control animals underwent sham surgery. Upon reperfusion, a 2 cm incision was cauterised along the ileal anti-mesenteric border. Exposed mucosal villi were visualised using an inverted intravital microscope (Olympus BX-81; Olympus). At 30 minutes reperfusion, 2×10^6^ CFSE labeled HPC-7 were administered intra-arterially. To examine the role of cytokines in mediating HSC recruitment, a segment of healthy small intestine, in which the mucosa was exposed, was lowered into a custom-built well filled with 1 ml of CXCL12, IL-1β, TNF-α (200 ng/ml; Peprotech) or PBS for 4 hours. The cytokine solution was replaced hourly. Subsequently, 2×10^6^ CFSE labeled HPC-7 were administered intra-arterially and monitored intravitally. This is a novel approach to study the role of cytokines in mediating SC homing in the gut.

### Intravital imaging of cremaster muscle

The testis was exposed through a scrotal incision and the cremaster muscle exteriorised, cleared of connective tissue, pinned open and continuously superfused with bicarbonate buffered saline. To induce ischemia, the cremasteric feeding arteriole was clamped for 30 minutes. 1×10^6^ HPC-7 were introduced via a femoral artery cannula post-reperfusion, with intravital observations made at 90 minutes. To examine the role of cytokines in mediating HPC-7 recruitment, the muscle was pre-treated with an intrascrotal injection of vehicle (200 µl), TNF-α (100 ng/200 µl) or IL-1β (12.5 ng/200 µl), with observations made 4 hours post-stimulus. In some mice, the muscle was treated topically with vehicle (1 ml) or CXCL12 (2 µg in 1 ml).

### Intravital microscopy data analysis

For analysis, one field of view (x10; 1.2×10^−1^ mm^2^) was selected randomly prior to cell administration. Images were obtained using an Olympus IX81 inverted microscope with a Photometrics CoolSNAP camera with an exposure time of 200 ms. Free flowing HPC-7 were classified as visible cells trafficking through the field of view, but non-adherent. HPC-7 rolling was determined by counting rolling cells on a 100 µm segment of post-capillary venule (PCV) for 60 seconds. 4 and was a distinct event from free flowing cells. HPC-7 were considered adherent if stationary for ≥30 seconds. At the end of cremaster experiments, all HPC-7 adherent within PCVs and capillaries within the entire cremaster (approximately 20 fields) were counted. At the end of intestinal experiments, images were obtained from an additional six fields to ensure events monitored in the pre-selected field were representative. Digital data was analyzed off-line (Slidebook, Intelligent Imaging Innovations, USA).

### Leukocyte recruitment

In mice undergoing intestinal IR injury, either 100 µl 0.9% saline, 2×10^6^ HPC-7 or 2×10^6^ CXCL12 pre-treated HPC-7 (100 ng/ml CXCL12, 1 hour) were administered systemically at 30 minutes post-reperfusion. 25 µl of 0.1% AcrO solution (Sigma-Aldrich) was injected at 45, 105, 165 and 225 minutes post-reperfusion. The intestines were prepared for imaging as described above. Leukocyte adhesion was counted at 60, 120, 180 and 240 minutes post-reperfusion by counting labeled cells from five random fields of view.

### Integrin clustering

2×10^6^ CTO-labelled HPC-7 were treated with 100 ng/ml CXCL12 (or vehicle control) for one hour at 37°C. Cells were PBS-washed and fixed in 5% neutral buffered formalin for one hour at room temperature. Following fixation, HPC-7 were washed in 2% PBSA, rested and then incubated with 1∶50 dilution of primary antibody (LEAF rat anti-mouse CD18 (GAME-46, Biolegend) or LEAF rat anti-mouse CD49d (R1-2, Biolegend) in 1% PBSA for one hour on ice. Cells were subsequently washed and then incubated with secondary antibody (Alexa 488 conjugated goat anti-rat, Invitrogen) at a dilution of 1∶100 for 30 minutes on ice. Cells were washed, allowed to settle onto lysine coated coverslips and mounted using Hydromount (National Diagnostics, Hessle, UK). Cells were imaged using scanning confocal microscopy (Leica Microsystems, Germany). Areas of clustering were identified using the “Find Maxima” plugin in ImageJ (NIH, NH, US). Maxima plots were produced by identifying regions of maximal intensity within control determined tolerance level to separate clusters from background.

### Statistics

For analysis of serial data, the area under the curve (AUC) was calculated for each experiment, the mean obtained for each group and values compared using an unpaired Student's t-test. This methodology is better suited to serial data as other statistical tests reduce statistical power by disregarding correlations between subjects [19]. Leukocyte adhesion following saline or cell administration was analysed by t-test at 4 hours. All remaining data was compared using an unpaired Student's t-test. Results were considered significant when p<0.05.

## Results

### HPC-7 adhesion is increased in IR injured intestine

**Figure 1:**
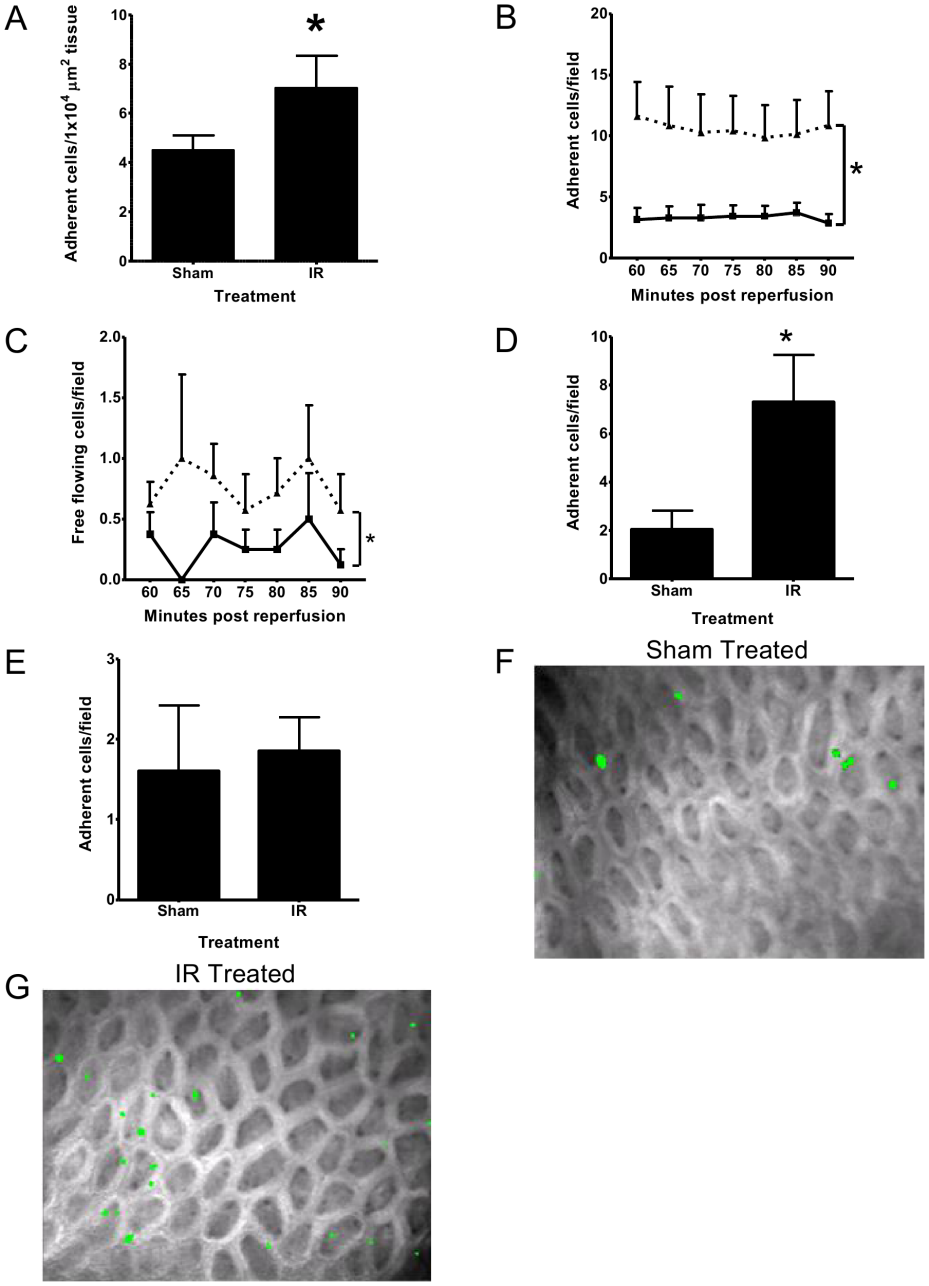
HPC-7 adhesion *in vitro* and *in vivo* is increased on IR injured intestine. (A) HPC-7 adhesion in vitro was raised on frozen ileal sections isolated from IR injured animals when compared to sham controls. Results represent mean adhesion per 1×10^4^ µm^2^ tissue area±SEM (n>4/group); * p<0.05. (B) HPC-7 adhesion in ileal mucosal microcirculation in vivo was also raised in animals subjected to IR injury (solid line: sham; dashed line: IR injury). (C) Free-flowing HPC-7 cells were increased in IR mice (solid line: sham; dashed line: IR injury). (D) IR injury enhanced HPC-7 adhesion within jejunal villi when quantitated ex vivo. (E) IR injury did not enhance HPC-7 adhesion to duodenum when quantitated ex vivo. Representative images of the villous microcirculation of the ileum in (F) sham and (G) IR animals are shown. Results are presented as mean adhesion±SEM (n≥7/group); * p<0.05. For frozen section work, data is expressed as mean adhesion per 1×10^4^ µm^2^ of tissue to control for area variation.

Significantly (p<0.05) more HPC-7 adhered to frozen tissue sections isolated from IR injured ileum compared to control sections (Figure 1a). Similar results were observed *in vivo* with significantly (p<0.05) more HPC-7 adherent within the intestinal microvasculature of ileum following IR injury compared to controls (AUC; sham: 18.44±4.61 vs IR: 62.71±17.12; Figure 1b). Numbers of free flowing cells in ileal mucosa were also significantly (p<0.05) higher in IR injured animals compared to controls (AUC; sham: 1.63±0.88 vs. IR: 4.71±1.32; Figure 1c). Additionally, *ex vivo* examination of jejunal (Figure 1d), but not duodenal mucosa (Figure 1e), revealed significantly (p<0.05) increased HPC-7 adhesion in IR injured animals compared to controls.

### HPC-7 administration does not reduce leukocyte adhesion in response to injury

**Figure 2:**
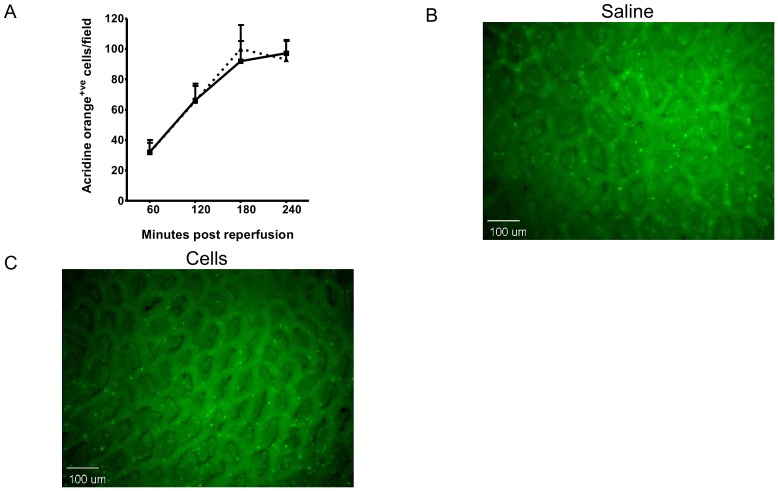
Recruited HPC-7 do not reduce leukocyte infiltration in IR injured intestine. (A) Leukocyte infiltration, analysed by AcrO staining, did not reduce in animals receiving 2×10^6^ HPC-7 cells at 30 minutes post-reperfusion when compared to IR injured animals receiving a saline bolus ie. no cells. Representive images from the ileum of IR treated animals receiving (B) saline or (C) 2×10^6^ HPC-7 are shown. Results are presented as mean cells per field±SEM.

IR injury was associated with increased numbers of adherent leukocytes over the 4 hour reperfusion duration. To determine whether recruited HPC-7 could modulate leukocyte adhesion following IR injury, endogenous leukocytes were labelled with acridine orange. Animals subsequently received either 100 µl 0.9% saline or 2×10^6^ HPC-7 at 30 minutes reperfusion. However, no difference in leukocyte adhesion at any time point during the 4 hour reperfusion period was noted between saline or cell treated animals (Figures 2a–c).

### HPC-7 and KSL cells express CD18 and CD49d

**Figure 3:**
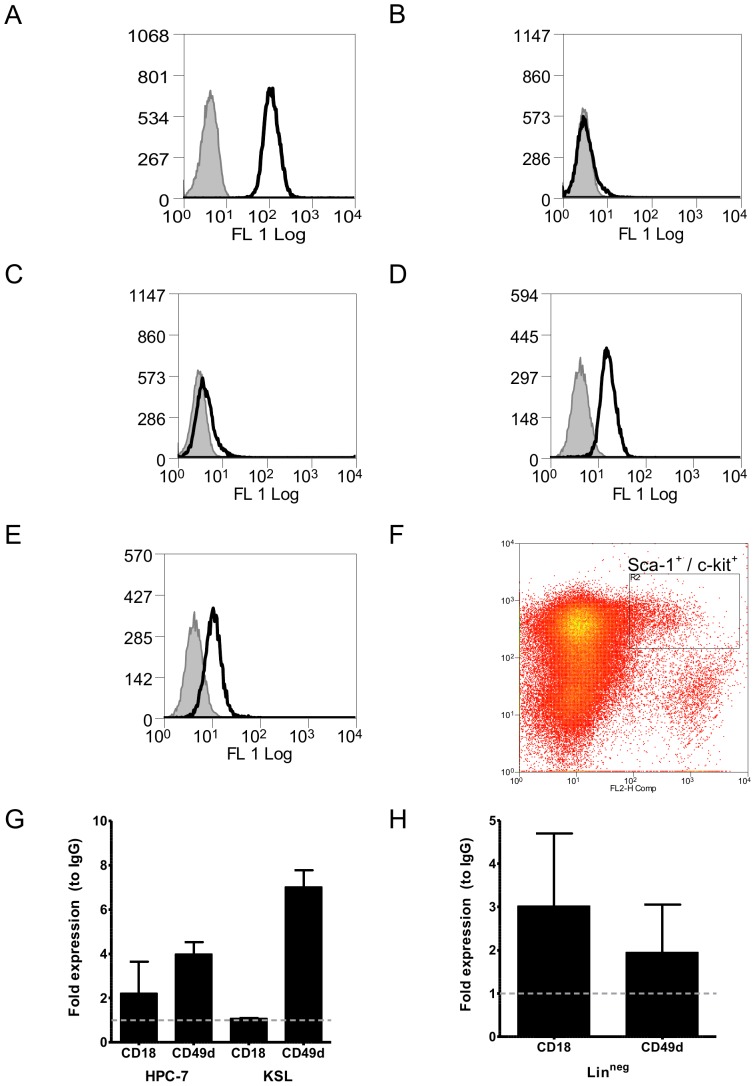
HPC-7 and KSL cells express CD18 and CD49d. HPC-7 cells express (A) CD11a, but not (B) CD11b or (C) CD11c. Flow cytometry also revealed expression of (D) CD18 and (E) CD49d on the surface of HPC-7. (F) Whole bone marrow was depleted for lineage postive cells and labelled for Sca-1, c-kit and either IgG, CD18 or CD49d. The gating used is shown in panel F (FL2: Sca-1; FL3: c-Kit). (G) KSL cells express cell surface CD49d, while CD18 could not be detected. (H) CD18 and CD49d could be identified by flow cytometry following cell permeabilization.

Flow cytometry revealed expression of CD11a (Figure 3a), but not CD11b (Figure 3b) or CD11c (Figure 3c) on HPC-7. In addition, flow cytometry revealed expression of CD18 (Figure 3d) and CD49d (Figure 3e) on HPC-7. To compare this profile to primary HSCs, adhesion molecule expression was assessed on c-Kit/Sca-1 labelled Lin^−^ cells (KSL cells). KSL cells expressed comparable levels of CD49d (Figure 3g). Expression of CD18 was not found on the surface of KSL cells (Figure 3g). To examine whether this was due to loss or internalisation of CD18, flow cytometry was performed on permeabilized cells. Following permeabilization, CD18 positivity could be detected in the KSL population (Figure 3h).

### HPC-7 adhesion to injured intestinal microcirculation is dependent on CD18 *in vivo*

**Figure 4:**
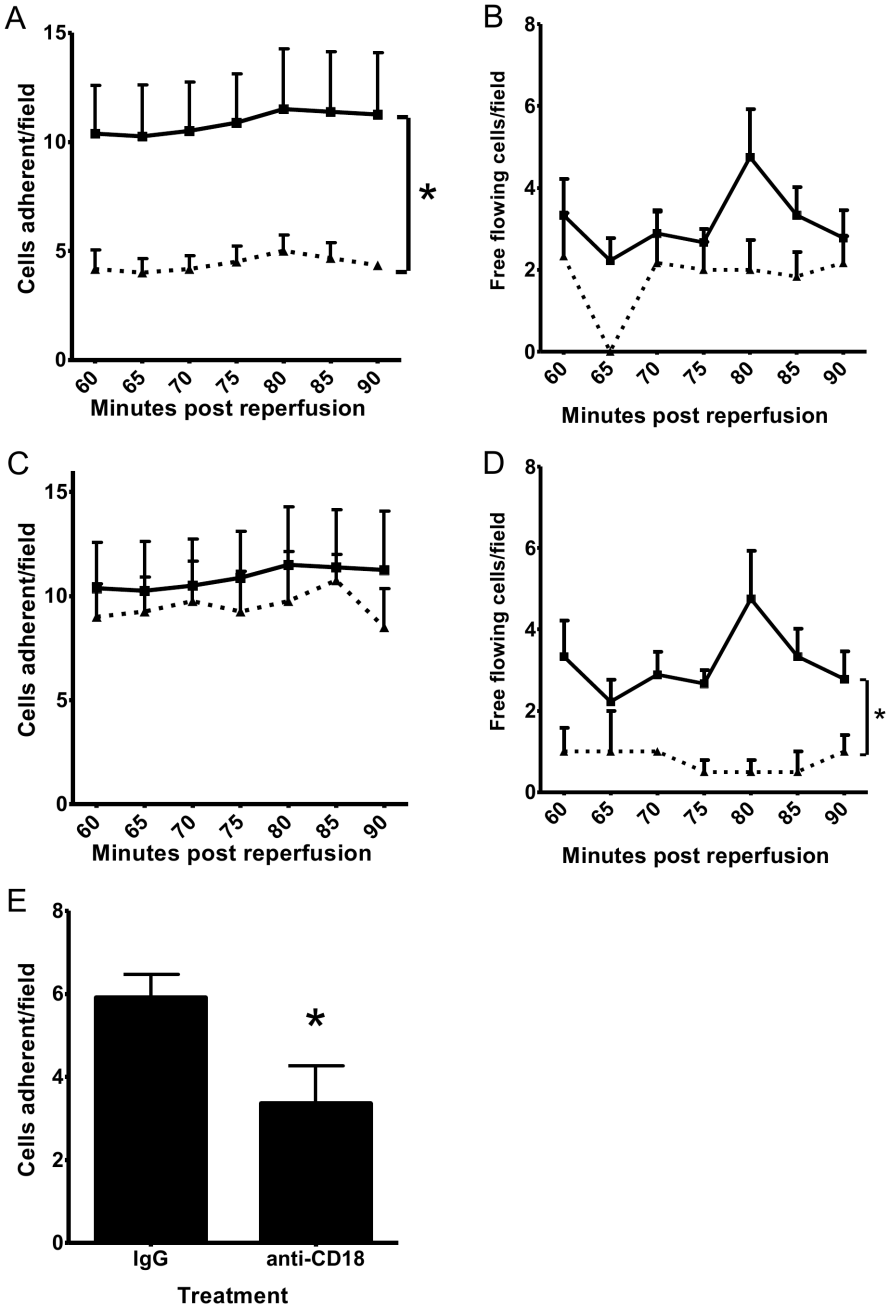
HPC-7 adhesion *in vivo* to intestinal microcirculation is dependent on CD18. (A) Pre-treatment of HPC-7 with an anti-CD18 antibody reduced adhesion in the IR injured gut microcirculation (solid line: IgG; dashed line: anti-CD18). (B) Pre-treatment with an anti-CD18 antibody did not significantly reduce free-flowing cells in the ileum (solid line: IgG; dashed line: anti-CD49d). (C) Pre-treatment of HPC-7 with anti-CD49d antibody did not reduce their adhesion in the IR injured gut microcirculation (solid line: IgG; dashed line: anti-CD49d). (D) Pre-treatment of HPC-7 with anti-CD49d antibody significantly reduced free flowing cells in the ileum (solid line: IgG; dashed line: anti-CD49d). (E) Treatment of HPC-7 with an anti-CD18 antibody reduced adhesion in the jejunum. Results are presented as mean adhesion per field±SEM (n≥4/group); * p<0.05.

HPC-7 adhesion following intestinal IR injury was significantly reduced by pre-treating cells with an anti-CD18 antibody (AUC: IgG: 65.31±14.63 vs anti-CD18: 27.21±1.79; p<0.05; Figure 4c). Pre-treating cells with an anti-CD18 antibody did not significantly reduce free-flowing HPC-7 in IR injury (AUC: IgG: 18.67±3.05 vs anti-CD18: 10.25±3.24; Figure 4d). However, adhesion was not reduced by pre-treating cells with an anti-CD49d antibody (AUC: IgG: 65.31±14.63 vs anti-CD49d: 57.5±10.15; Figure 4e). Pre-treating cells with an anti-CD49d antibody significantly reduced free-flowing HPC-7 in IR injury (AUC: IgG: 18.67±3.05 vs anti-CD49d: 4.5±1.24; p<0.05; Figure 4g). Jejunual HPC-7 adhesion, determined on frozen tissue assays, was also significantly reduced with an anti-CD18 antibody (p<0.05; Figure 4h).

### HPC-7 adhesion is increased in injured muscle microcirculation and is predominantly dependent on CD49d

**Figure 5:**
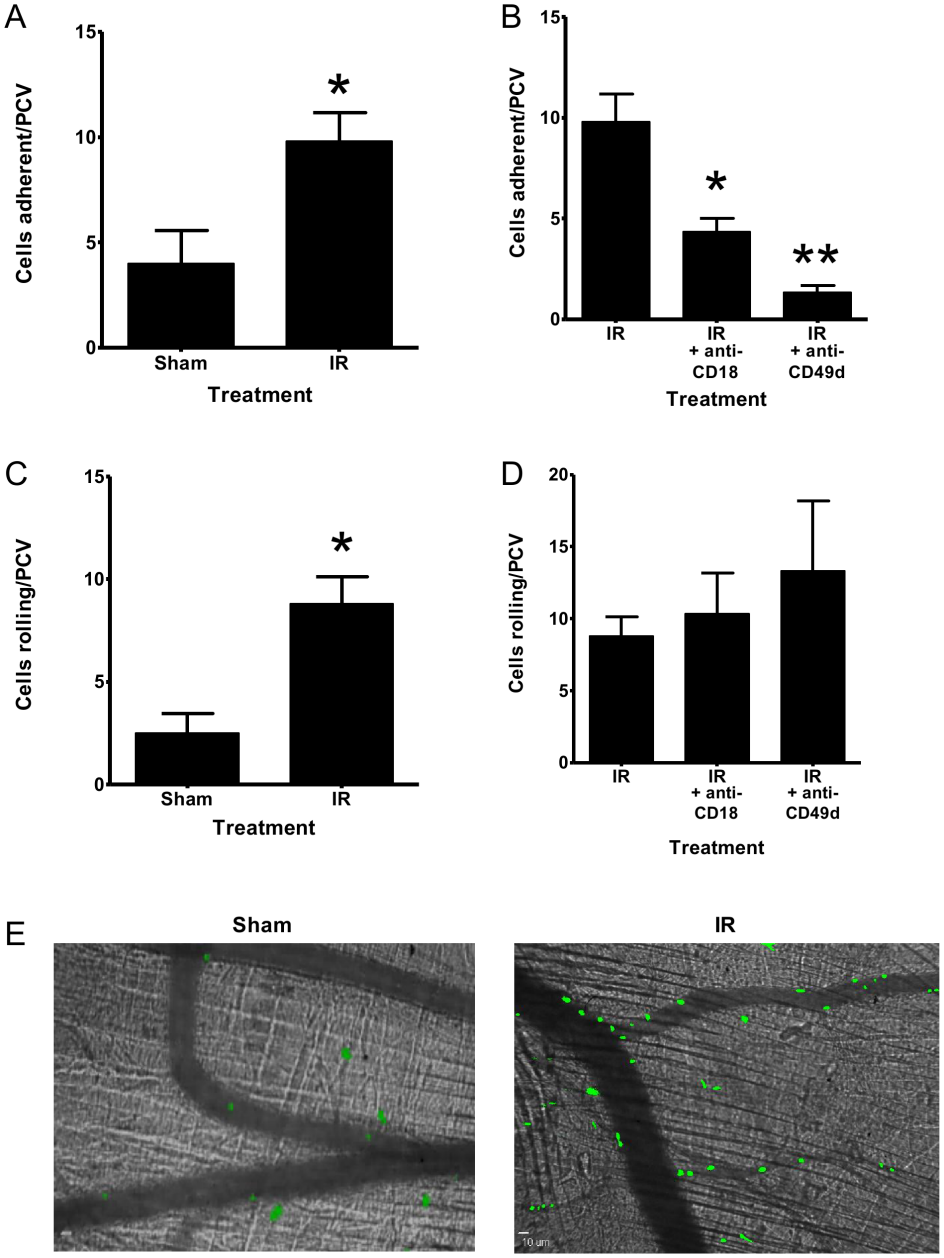
HPC-7 adhesion *in vivo* is increased in IR injured muscle and is dependent on CD49d. (A) HPC-7 adhesion to cremaster PCVs was enhanced following IR injury. (B) Pre-treating HPC-7 with an anti-CD18 or anti-CD49d blocking antibody reduced HPC-7 adhesion. (C) IR injury enhanced HPC-7 rolling in PCVs. (D) Blocking with anti-CD18 or anti-CD49d antibodies did not enhance rolling above that seen with IR injury alone. (E) Representative images (x10 magnification) of HPC-7 adhesion in sham and IR injury are shown. Results are presented as mean adhesion±SEM (n≥3/group); A/C: * p<0.05, IR vs Sham; B: * p<0.05, ** p<0.01, IR vs antibody blocked cells.

HPC-7 adhesion was significantly (p<0.05; Figures 5a, e) increased in PCVs following muscle IR injury compared to controls (Figures 5a, e). Adhesion within PCVs was significantly reduced by pre-treating HPC-7 with anti-CD18 (p<0.05; Figure 5b) or anti-CD49d antibody (p<0.01; Figure 5b).This reduction was greatest with blocking of CD49d, with only a partial reduction observed when blocking CD18. HPC-7 rolling was significantly (p<0.05; Figure 5d) increased in PCVs following IR injury compared to controls which was not altered with either anti-CD18 or anti-CD49d. Representative images of HPC-7 adhesion in sham and IR injured mice is shown (Figure 5e).

### Topical treatment of healthy intestine with cytokines increases HPC-7 adhesion *in vivo*

**Figure 6:**
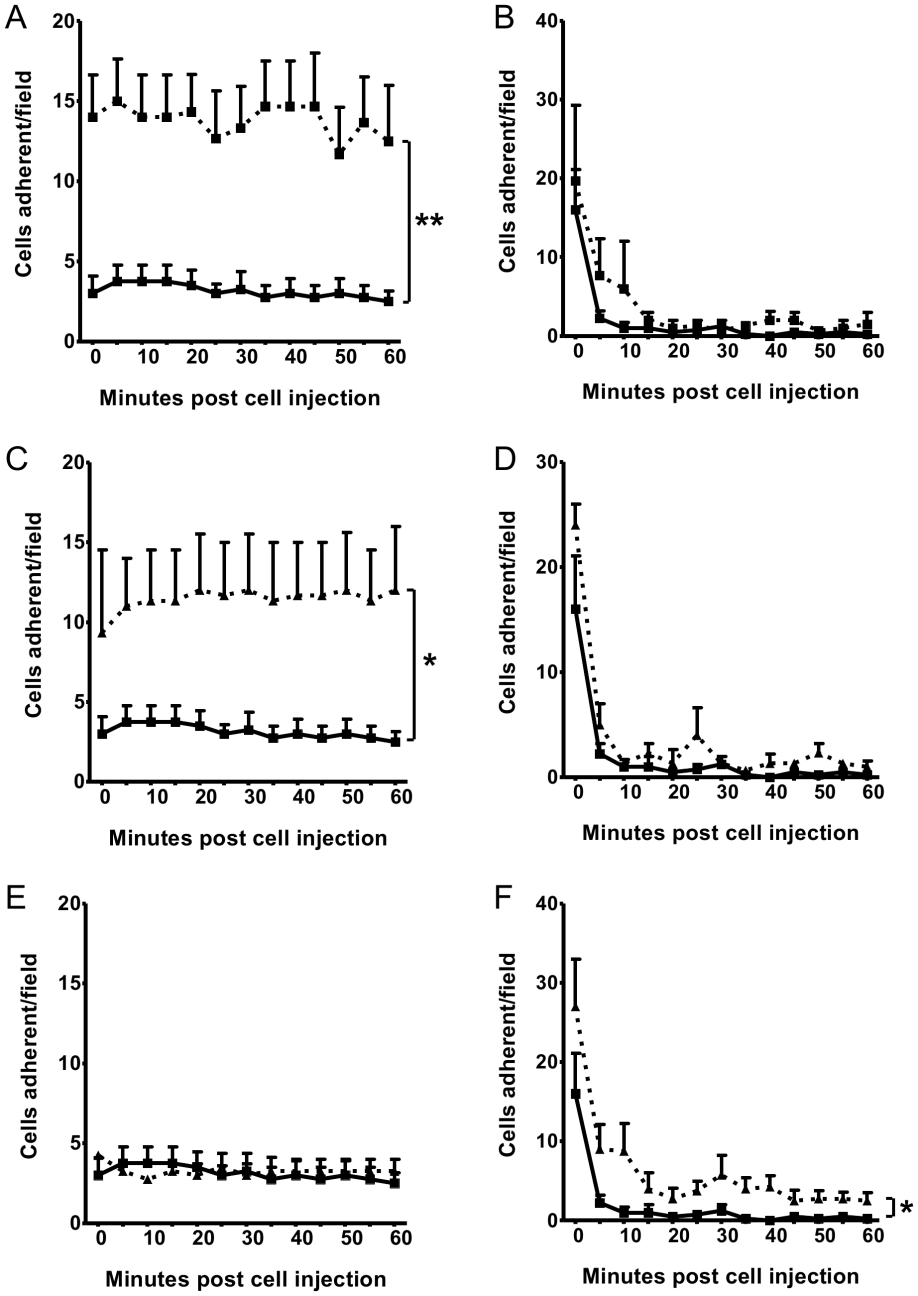
Topical treatment of the small intestine with cytokines increases HPC-7 adhesion *in vivo*. (A) Mucosal treatment with TNF-α significantly enhanced adhesion of HPC-7 cells *in vivo*. (B) No increase in free-flowing HPC-7 was observed following topical treatment of the gut with TNF-α. (C) Mucosal treatment with IL-1β significantly enhanced adhesion of HPC-7 cells *in vivo.* (D) No increase in freeflowing HPC-7 was observed following topical treatment of the gut with IL-1β. (E) No increase in adhesion was observed following topical treatment of the gut with CXCL12. (F) Significantly increased numbers of HPC-7 were observed free-flowing through the gut following topical CXCL12 treatment. Results are represented as mean cells adherent/field±SEM (n≥3/group); * p<0.01, ** p<0.01, PBS topical treatment vs Cytokine topical treatment.

Topical exposure of non-injured ileal mucosa to TNF-α (p<0.01; Figure 6a) or IL-1β (p<0.05; Figure 6c), significantly enhanced the adhesion of injected HPC-7 cells *in vivo*. Enhanced adhesion of HPC-7 cells was not observed following topical treatment of the ileum with CXCL12 (Figure 6e). In contrast, neither topical treatment of the ileum with TNF-α (Figure 6b) or IL-1β (Figure 6d) enhanced the number of cells that could be identified freely flowing or trafficking through the ileum. Conversely, topical treatment of the ileum with CXCL12 significantly enhanced the number of cells that could be identified freely flowing through the ileum (p<0.05; Figure 6f).

### TNF-α and IL-1β significantly enhance adhesion in the cremaster but this is not augmented with topical treatment of the cremaster with CXCL12

**Figure 7:**
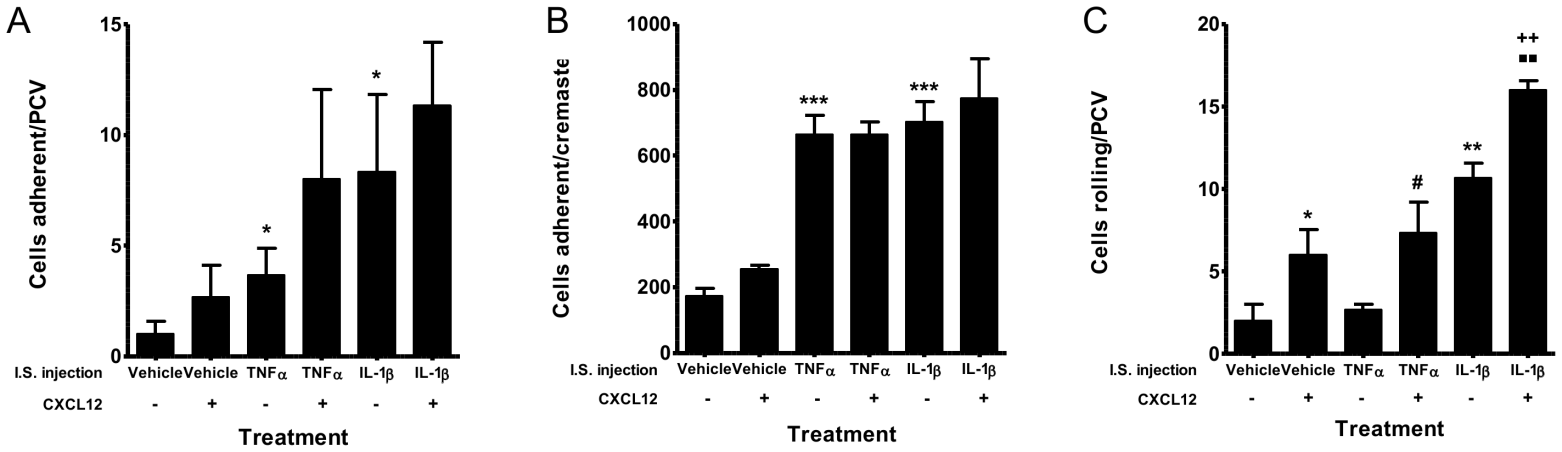
TNF-α and IL-1β significantly enhance adhesion in the cremaster but this is not augmented with topical treatment of the cremaster with CXCL12. Cremasteric exposure to only TNF-α and IL-1β promoted HPC-7 adhesion when monitored in (A) individual PCVs or (B) the entire cremaster. This adhesion was not increased further in the presence of CXCL12. (C) Rolling was affected by IL-1β and CXCL12 alone and was enhanced further in the presence of both together. Results are presented as mean cells adherent per PCV, mean rolling cells per PCV and mean cells adherent per cremaster±SEM (n = 3/group); * p<0.05, ** p<0.01, *** p<0.001 (vs vehicle with no CXCL12); #p<0.05 (vs TNF-α with no CXCL12); ++p<0.01 (vs vehicle with CXCL12); ▪▪ p<0.01 (vs IL-1β with no CXCL12).

Similarly, HPC-7 adhesion within muscle was increased following intrascrotal injection of TNF-α and IL-1β, both in PCVs (p<0.05; Figure 7a) and when adhesion was quantitated in the whole cremaster (p<0.001; Figure 7b). HPC-7 adherent within the whole cremaster were observed primarily within the capillaries. Topical CXCL12 did not increase HPC-7 adhesion either in cremasteric PCVs or within the whole cremaster (Figures 7a, b). To determine whether dual exposure to CXCL12 and either TNF-α or IL-1β could synergistically enhance adhesion, the cremaster was treated topically with CXCL12 and intrascrotally with TNF-α or IL-1β. However, no enhancement of adhesion above that seen with TNF-α or IL-1β alone was observed with CXCL12 co-treatment (Figures 7a, b). TNF-α did not increase PCV rolling whereas this event was significantly increased following intrascrotal injection of IL-1β (p<0.01; Figure 7c) and CXCL12 (p<0.05; Figure 7c). CXCL12 in addition to TNF-α did not enhance rolling above that seen with CXCL12 alone (Figure 7c). CXCL12 in addition to IL-1β significantly enhanced PCV rolling above that seen with IL-1β and CXCL12 alone (p<0.01; Figure 7c).

### Pre-treating HSCs with cytokines enhanced their adhesion to endothelium *in vitro*, IR injured intestine *in vivo* and reduces leukocyte adhesion post-IR injury

**Figure 8:**
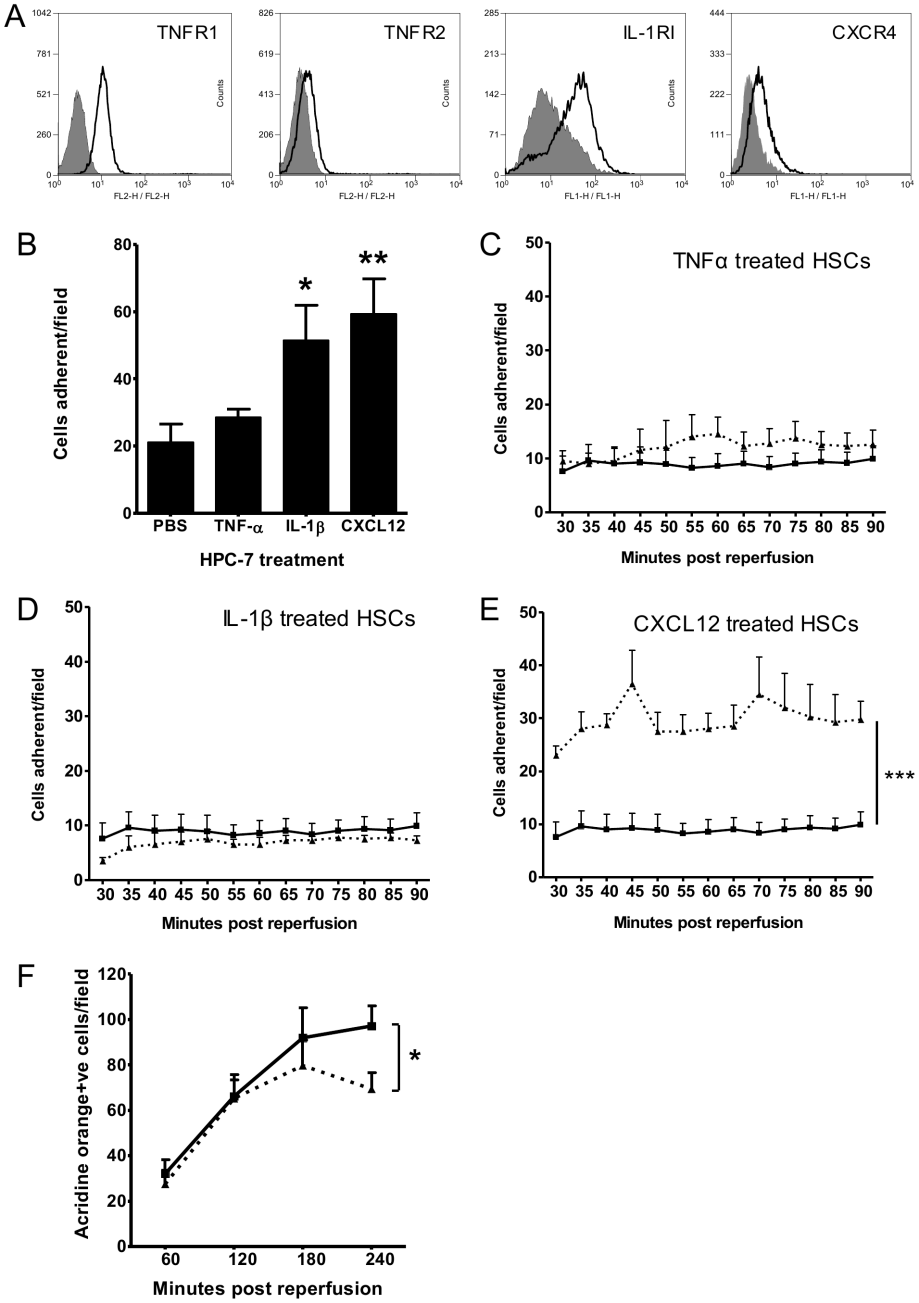
HPC-7 pre-treatment with CXCL12 enhances their adhesion on colonic endothelium *in vitro*, IR injured intestine *in vivo* and reduces leukocyte infiltration post-IR injury. (A) HPC-7 express the cytokine receptors TNFR1, TNFR2, IL-1RI and CXCR4. (B) Pre-treatment of HPC-7 with 100 ng/ml IL-1β or 100 ng/ml CXCL12 enhances adhesion of HPC-7 on TNF-α activated colonic endothelium in vitro. Pre-treatment of HPC-7 with (C) 100 ng/ml TNF-α or (D) 100 ng/ml IL-1β does not enhance intestinal HPC-7 adhesion following IR injury in vivo. (E) Pre-treatment of HPC-7 with 100 ng/ml CXCL12 enhances adhesion in the intestine following IR injury in vivo. (F) Leukocyte infiltration, analysed by AcrO staining, was reduced at four hours in animals receiving 2×10^6^ CXCL12 pre-treated HPC-7 cells at 30 minutes post-reperfusion when compared to IR animals receiving a saline bolus. Results are presented as mean adherent cells per field±SEM (Figure B, n = 3; Figures C-E, n≥3; Figure F, n≥12); * p<0.05, ** p<0.01, *** p<0.001 vs control).

Flow cytometry demonstrated that HPC-7 express TNFR1/2, IL-1RI, and CXCR4, receptors for TNF-α, IL-1β and CXCL12 respectively (Figure 8a). Pre-treatment of HPC-7 with IL-1β (p<0.01; Figure 8b) or CXCL12 (p<0.05; Figure 8b) significantly increased adhesion to TNF-α activated murine colonic endothelium compared to controls. Only pre-treatment of HPC-7 with CXCL12 significantly (p<0.001; Figure 8b) enhanced intestinal HPC-7 adhesion following IR injury *in vivo* (Figures 8e). Pre-treatment with neither TNF-α (Figure 8c) nor IL-1β (Figure 8d) enhanced intestinal HPC-7 adhesion following IR injury *in vivo*. Interestingly, enhancing HPC-7 adhesion by pre-treating them with CXCL12 significantly (p<0.05) lowered leukocyte adhesion at 4 hours compared to animals receiving a saline control (Figure 8f).

### Pre-treating HSCs with cytokines enhances their binding to endothelial counterligands and enhances surface integrin clustering

**Figure 9:**
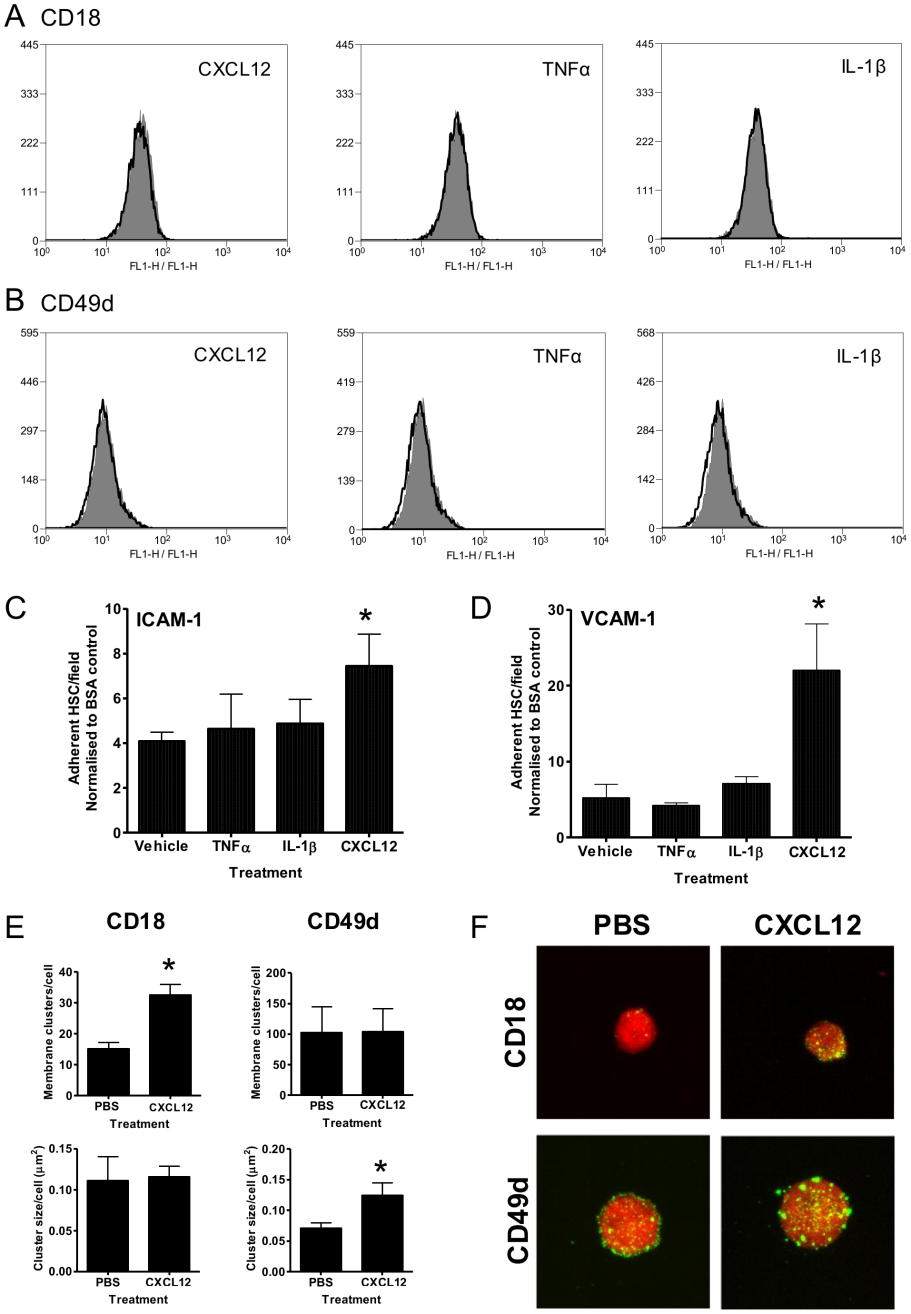
Pre-treating HPC-7 with CXCL12 increases their binding to endothelial counterligands VCAM-1 and ICAM-1, but does not increase levels of surface integrin expression. No up-regulation of (A) CD18 or (B) CD49d is identifiable by flow cytometry following treatment of HPC-7 with CXCL12, TNF-α or IL-1β. Pre-treatment of HPC-7 with CXCL12 enhances HPC-7 adhesion on (C) ICAM-1 and (D) VCAM-1. Treating HPC-7 with CXCL12 for an hour significantly enhances the (E, top left) number, but not size (E, bottom left) of membrane CD18 clusters when compared to PBS controls. Conversely, treating HPC-7 with CXCL12 for an hour significantly enhances the size (E, bottom right) but not number (E, top right) of CD49d clusters on the surface of HPC-7. Representative images are shown in (F). Results are presented as representative flow cytometry plots (Figure A, B, representative plot from n = 3), mean adherent HPC-7/per field, normalized to adhesion on a BSA control±SEM (Figure C and D, n = 3/group) and cluster count/size as analysed by ImageJ (Figure E, n = 3/group); * p<0.05 (vs vehicle treated).

No up-regulation of CD18 (Figure 9a) or CD49d (Figure 9b) was observed when HPC-7 were pre-treated with TNF-α, IL-1β or CXCL12. Pre-treating HPC-7 with IL-1β or TNF-α did not enhance HPC-7 adhesion to ICAM-1 or VCAM-1 coated surfaces (Figures 9c, d). However, CXCL12 pre-treatment significantly enhanced adhesion to both ICAM-1 and VCAM-1 (p<0.05; Figures 9b, c). Treatment with CXCL12 significantly increased the number of CD18 clusters on the HPC-7 cell surface without enhancing the size of each individual cluster (p<0.05; Figures 9e, f). Conversely, CXCL12 did not significantly enhance the number of CD49d clusters, but did significantly increase their size on the cell surface (p<0.05; Figure 9e, f).

## Discussion

Although evidence suggests HSCs may be beneficial for degenerative and ischemic disorders, their efficacy is likely to be proportional to the degree of cell adhesion achieved. Enhancing the effectiveness of reparative processes may therefore depend on identifying adhesive mechanisms that underpin HSC trafficking. This novel *in vivo* study initially demonstrated that HPC-7, an accepted HSC line, could be recruited to both murine villous capillaries and muscle venules following an acute injury. We have shown previously that HPC-7 adhesion within hepatic microcirculation is CD49d dependent and CD18 independent [10]. However, we show here that while both CD49d and CD18 are involved in HPC-7 adhesion within injured muscle, recruitment to gut was only dependent upon CD18. This suggests that site specific adhesive mechanisms exist for HPC-7 recruitment, although there is overlap between the mechanisms used between some tissues. In muscle, we observed HPC-7 rolling followed by firm adhesion, suggesting HPC-7 are able to utilise a similar adhesion paradigm to that described for mature leukocytes. Although local healthy tissue pre-treatment with TNF-α and IL-1β was capable of enhancing HPC-7 homing to both vascular beds, tissue treatment with CXCL12 did not enhance HPC-7 adhesion. However, pre-treating HPC-7 with CXCL12 did enhance their adhesion within injured gut, most likely achieved through CXCL12 mediated effects on CD18. Importantly, leukocyte adhesion within the IR injured intestine was reduced significantly following administration of CXCL12 pre-treated HPC-7. No reduction in leukocyte adhesion was noted in animals receiving no cells or control treated cells.

The observed increases in adhesion and free-flowing cells are unlikely to result from post-ischemic increases in blood flow to injured tissues. Following brief periods of ischemia, reactive hyperaemia is a well established mechanism by which blood flow to a previously ischemic tissue is enhanced upon reperfusion. However, following more significant periods of ischemia, tissue blood flow during reperfusion is reduced relative to pre-ischemic levels. A number of studies demonstrate that in the small intestine, blood flow in the reperfusion phase of IR is reduced from baseline and progressively worsens during reperfusion over the acute term [20]–[22]. Studies suggest that this flow can remain decreased up to 24 hours post-reperfusion [23]. Using laser speckle contrast imaging, unpublished studies from our lab have found that colonic blood flow following ischemia is reduced and fails to improve over at least the first two hours of reperfusion (data not shown). We have obtained similar results following IR in the kidney (White *et al*, manuscript submitted). Collectively, this suggests that the increased HPC-7 adhesion observed is not due to increased blood flow to the injured bowel. The increased number of free flowing cells in IR injured tissue may be explained by a reduction in the speed of these cells as they traffic through the injured tissue, and we have evidence of this happening in our renal studies (unpublished data).

It is possible that the differential dependency on integrins between tissues is linked to the expression of endothelial counter-receptors. A major determinant of the relative contribution of CD29-based and CD18-based integrins to leukocyte recruitment in different pathologies is the density of endothelial VCAM-1 and ICAM-1 expression [24]. Interestingly, constitutive expression of ICAM-1 in the gut is much higher than VCAM-1 [25], which may account for the differential roles of CD49d and CD18 in this tissue. Although increased expression of VCAM-1 can be observed on IR injured intestinal tissue sections [26], the time course involved may preclude involvement of *de novo* VCAM-1 expression in the current study. Following activation of ECs with cytokines, surface VCAM-1 appears at around 4 hours and peaks at 12 hours [27]. It is possible that HPC-7 recruitment in the current study takes place prior to significant VCAM-1 up-regulation. It is interesting that CD49d plays an important role in muscle given the low basal levels of VCAM-1 and the time course of up-regulation. However, IR injury may induce a greater or more rapid increase in cremasteric VCAM-1 than in gut. Certainly, endothelial activation with TNF-α, IL-1β or bacterial endotoxins can differentially increase levels of ICAM-1 and VCAM-1 expression on ECs derived from different sources [24].

Following intestinal IR injury, pro-inflammatory cytokines are released into the local circulation, typically preceding *de novo* ICAM-1/VCAM-1 up-regulation [28], [29]. Indeed, TNF-α is a key mediator of intestinal inflammation and is elevated in patients with IBD [30]. This study provides novel data, generated by exposing healthy gut to TNF-α or IL-1β, demonstrating that either of these inflammatory cytokines may act as a recruitment mechanism for HPC-7 during the early phase of IR injury and this may mediate recruitment prior to endothelial adhesion molecule up-regulation. In contrast to the adhesion molecules required, this suggests that there is no site-specificity with regards the cytokines that can lead to HPC-7 adhesion.

Since HSCs are exposed to inflammatory cytokines whilst trafficking through injured tissues, we hypothesised that pre-treating HPC-7 prior to infusion would enhance their retention within injured gut. However, TNF-α pre-treatment did not enhance adhesion to ECs, ICAM-1/VCAM-1 or *in vivo*. Although TNF-α stimulation of neutrophils up-regulates CD11b/CD18 expression [31], no up-regulation of CD18 was observed when HPC-7 were TNF-α stimulated. This suggests that TNF-α mediated HPC-7 adhesion most likely occurs via endothelial activation, independent of direct effects on HPC-7. The role of TNF-α in an injured microenvironment may not just be limited to promoting SC adhesion. It is critical in stimulating MSCs to secrete beneficial growth factors in order to promote repair [32]. This paracrine release of growth factors has also been suggested as a mechanism by which HSCs may exert beneficial effects [33]. IL-1β is a pro-inflammatory cytokine strongly implicated in intestinal inflammation and is a potent mediator of neutrophil recruitment [34]. Furthermore, IL-1β is important for proliferation and differentiation of HSCs during haematopoiesis [35]. The current study demonstrates that IL-1β is capable of inducing HPC-7 adhesion *in vivo* in two different tissue beds. Similarly to TNF-α, pre-treatment of HPC-7 with IL-1β did not enhance adhesion *in vivo*.

CXCL12 is strongly associated with HSC homing to BM and sites of peripheral injury [36], [37]. However, a direct role for CXCL12 in mediating HSC adhesion within intestinal microvasculature is unknown. Interestingly, we demonstrate that exposure of healthy intestinal mucosa or muscle to CXCL12 did not promote HPC-7 adhesion. Previous studies have demonstrated that CXCL12 alone is insufficient to recruit SCs to ischemic heart in the absence of injury [38] or promote HSC adhesion to ECs without TNF-α stimulation [39]. Whilst TNF-α and IL-1β have well defined activating effects on ECs [40], there is little evidence to suggest that CXCL12 causes endothelial activation. This may explain why topical CXCL12 alone was unable to enhance HPC-7 recruitment. Interestingly, although topical CXCL12 treatment did not enhance HPC-7 adhesion, there was a significant increase in free-flowing HPC-7 in treated tissues. This is likely to result from a reduction in the speed of cells trafficking through tissues. Endothelial cells are able to present exogenous CXCL12 bound to heparin sulphate proteoglycans (HSPGs) [41]. In addition, immobilized CXCL12 is able to promote tethering and increase resistance to shear flow of human HSCs on VCAM-1 coated surfaces *in vitro* [39]. In our models, exogenously administered CXCL12 may be binding to endothelial HSPGs and promoting transient tethering of trafficking HSCs.

Interestingly, only pre-treatment with CXCL12 enhanced HPC-7 adhesion both *in vitro* and *in vivo*. This provides novel evidence that HSC adhesion within injured gut is a modulatable event and that recruitment achieved by injury is not maximal. The increased adhesiveness appeared to be mediated by effects on the integrin sub-unit CD18, as demonstrated by enhanced HPC-7 binding to immobilised ICAM-1. This enhanced adhesion did not result from increased surface expression of integrins but may partially be due to CXCL12 mediated increases in the number of identifiable CD18 clusters on the HPC-7 surface. It has previously been shown that CD18 clustering alone, independent of increased expression, can enhance integrin mediated adhesion of cells under flow conditions [42]. Interestingly, CXCL12 did not enhance the number of identifiable CD49d clusters on the HPC-7 surface, but did increase the cluster size, representing increased CD49d localisation and an increased density of VCAM-1 ligands. Since CXCL12 also increased adhesion to VCAM-1, it is possible that this pre-treatment strategy may be equally effective at increasing HSC retention within organs in which recruitment depends upon the CD49d/CD29 integrins, such as the liver [10]. Attempts have been made to enhance SC recruitment by increasing local concentrations of CXCL12 within ischemic tissue using plasmids encoding for CXCL12 [43]. However, the delivery of vectors to allow for targeted expression of chemokines has been challenging, particularly in the clinical setting. The current study offers a more clinically appealing approach since it is non-invasive and does not require introduction of genetic material.

Although previous studies have demonstrated a benefit for BM-derived SCs for intestinal disorders [3], [44], the mechanisms by which repair is implemented remain unclear. Amelioration of injury without SCs needing to actually engraft the tissue has been demonstrated [45], suggesting paracrine modulation of inflammation by recruited SCs is possible [46]. Although immunomodulation has been demonstrated for BM-derived mesenchymal SCs, evidence for HSCs having similar effects is less well described. Since leukocytes are key players in mediating intestinal IR injury [47], we determined whether HPC-7 exerted an anti-inflammatory effect. Interestingly, our novel data demonstrated that administration of naïve HPC-7, recruited by the presence of the injury alone, did not affect intestinal leukocyte numbers at any time point post-reperfusion. However, CXCL12 pre-treatment, which enhanced HPC-7 recruitment, reduced leukocyte infiltration at 4 hours post-reperfusion. This suggests increased HSC presence may be required in order for inflammation to be modulated. It is also possible that the reduced leukocyte infiltration is brought about by CXCL12 ‘priming’ or activating anti-inflammatory functions of HSCs. Indeed, recent studies have shown that un-stimulated MSCs are incapable of immunosupression and that this function of MSCs is only initiated following treatment with inflammatory cytokines [47]. However, we have preliminary evidence from intravital studies in IR injured mouse kidney that HPC-7 can reduce leukocyte adhesion without the need for any pre-stimulation with CXCL12. This may be due to the interesting observation that strikingly higher numbers of HPC-7 adhere in injured renal peritubular micovessels than were observed in the gut. This suggests that simply enhancing the local presence of HPC-7 can mediate a greater benefit effect.

In conclusion, our novel data highlights the integrin sub-unit, CD18, as a critical component of HPC-7 adhesion within injured intestinal microvasculature *in vivo*. The selective use of specific integrins in HSC homing may be exploited therapeutically to ensure that systemically administered HSCs are delivered to injury sites more efficiently and not entrapped within non-injured sites. Importantly, this study provides novel data that a greater local presence of HPC-7 modulates inflammation, underscoring the need to identify strategies that enhance SC homing. Increasing the adhesive ability of surface integrins through non-genetically engineered approaches has translational potential for use clinically to improve HSC recruitment. These results suggest CXCL12 pre-treatment has potential to be used clinically as an adjuvant therapy to enhance HSC recruitment. Such strategies may enhance the therapeutic benefit of HSCs through acceleration of tissue recovery and thus improve the effectiveness of cellular therapy. The current data may therefore help in the design of future SC based trials for gastrointestinal disorders.
